# Hydrothermal Synthesis of Biomass-Derived Magnetic Carbon Composites for Adsorption and Catalysis

**DOI:** 10.1021/acsomega.1c05116

**Published:** 2021-11-24

**Authors:** Gareth Davies, James McGregor

**Affiliations:** Department of Chemical and Biological Engineering, University of Sheffield, Mappin Street, Sheffield S1 3JD, U.K.

## Abstract

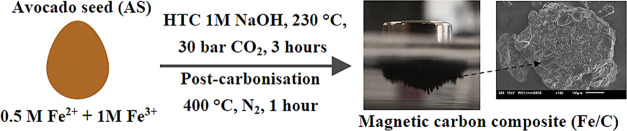

The synthesis of magnetic iron–carbon composites (Fe/C) from waste avocado seeds via hydrothermal carbonization (HTC) has been demonstrated for the first time. These materials are shown to be effective in adsorption and catalytic applications, with performances comparable to or higher than materials produced through conventional processing routes. Avocado seeds have been processed in high-temperature water (230 °C) at elevated pressure (30 bar at room temperature) in the presence of iron nitrate and iron sulfate, in a process mimicking natural coalification. Characterization of the synthesized material has been carried out by X-ray diffraction (XRD), atomic absorption spectroscopy (AAS), X-ray fluorescence (XRF), X-ray photoelectron spectroscopy (XPS), inductively coupled plasma-optical emission spectrometry (ICP-OES), Fourier-transform infrared spectroscopy (FT-IR), magnetometry, and through surface area measurements. The supported iron particles are observed to be predominately magnetite, with an oxidized hematite surface region. The presence of iron catalyzes the formation of an extended, ordered polymeric structure in the avocado seed-derived carbon. The magnetic Fe/C has been demonstrated as an adsorbent for environmental wastewater treatment using methylene blue and indigo carmine. Kinetic analysis suggests that the adsorbates are chemisorbed, with the positive surface charge of Fe/C being preferential for indigo carmine adsorption (49 mg g^–1^). Additionally, Fe/C has been evaluated as a heterogeneous catalyst for the hydroalkoxylation of phenylacetylene with ethylene glycol to 2-benzyl-1,3-dioxolane. Product yields of 45% are obtained, with 100% regioselectivity to the formed isomer. The solid catalyst has the advantages of being prepared from a waste material and of easy removal after reaction via magnetic separation. These developments provide opportunities to produce carbon-based materials for a variety of high-value applications, potentially also including energy storage and biopharmaceuticals, from a wide range of lignocellulosic biomass feedstocks.

## 1 Introduction

Climate change, environmental pollution, decreasing fossil fuel reserves, and overpopulation are global grand challenges requiring effective scientific and political solutions.^1^ To tackle these challenges, it is imperative that sustainable processes for waste management and valorization are developed and implemented. The majority of the estimated 2 billion tonnes of municipal solid waste (MSW), which is produced globally each year is sent to landfills or incinerated.^2^ There are many potential alternative processing methods, which yield value-added products from MSW, such as anaerobic digestion, composting, pyrolysis, fermentation, and hydrothermal processing.^3,4^

Among these, hydrothermal processing and, in particular, hydrothermal carbonization (HTC), present significant opportunities to valorize organic wastes. HTC is a thermochemical conversion technology, which operates at low processing temperatures (180–260 °C) with sufficient pressure to maintain the water in the liquid phase (<50 bar). It has the further advantage of short processing times (30 min to 3 days).^5−8^ HTC simulates natural coalification and produces a solid material with increased energy density and carbon content relative to the initial feedstock and with decreased oxygen and volatile content. This solid is usually referred to as hydrochar.^8−11^ HTC can be applied to a variety of different waste feedstocks, including biomass, sewage sludge, and waste plastics. There is no need for pretreatment of the feedstock, thereby eliminating the need for costly predrying processes.^8,10,12,13^ This facilitates the treatment of high moisture content feedstocks such as MSW or biomass. The latter typically has a moisture content ranging from 0.5 to 1 kg of water per kg after oven-drying. Thermal drying costs of such moisture-rich materials are ∼1.64 MJ/kg_H_2_O_, equivalent to up to 11% of the energy content of lignite coal.^14^ As-synthesized hydrochar has properties approaching the characteristics of low-rank coals, with previous literature focusing on HTC of MSW for solid fuel production.^9,15−17^ However, HTC has also more recently been used to synthesize high-value carbon materials for applications in catalysis, surface adsorption, and energy storage.^11,18−21^ Additionally, post-synthesis treatments can upgrade hydrochar to yield a material with enhanced physicochemical characteristics for high-value applications.^22−24^

In the present study, avocado seeds (AS) have been investigated as a lignocellulosic feedstock for HTC. AS are a waste product from guacamole production in addition to the manufacture of sauces, oils, and frozen products.^25−27^ This is a rapidly growing industry, with the global production of avocados forecast to triple by 2030 as compared to 2010 values.^28^ This estimates an annual production of 12 Mt, generating ∼2 Mt of waste seeds. AS are only rarely used as animal feed because of their unpleasant taste and low nutrient content; hence, large amounts are disposed of to landfills. Additionally, AS present an unusual composition for waste biomass, with a high hemicellulose:cellulose ratio; e.g., Lin et al. have previously reported a ratio of ∼9:1 (mass basis).^29^ In contrast, more commonly investigated agricultural residues such as sugarcane bagasse present a higher content of cellulose than hemicellulose.^30,31^ It is therefore anticipated that in HTC of AS, the dominant conversion route will be carboninzation of soluble hemicelluloses. This therefore serves as an exemplar for the investigation of hemicellulose-rich waste streams and is, to the best knowledge of the authors, the first investigation of AS in hydrothermal processing.^25,32^

Iron oxide nanomaterials have applications in a wide variety of areas, including adsorption, heterogeneous catalysis, photocatalysis, immobilization, and biopharmaceuticals.^33,34^ The synthesis conditions employed to produce such materials can give precise control over their surface functionality. Furthermore, they can exhibit ferrimagnetism, high biodegradability, and low toxicity. Iron oxide nanomaterials can be produced using many different procedures, including sol–gel, oxidation, co-precipitation, hydrothermal, aerosol, supercritical, and microbial syntheses. Previous research has shown the potential to produce functional iron oxide materials supported on or encapsulated within hydrochar. For example, carbon-encapsulated nanoparticles have been produced using glucose as the carbon source.^35,36^ Other work has focused on post-synthesis modification of hydrochars produced from waste lignocellulosic materials through impregnation with an appropriate iron precursor.^37−39^ The latter approach typically requires pyrolysis at temperatures in excess of 600 °C to introduce porosity into carbon. Rattanachueskul et al. have demonstrated the conversion of sugarcane bagasse and an iron precursor into magnetic carbon composites via a one-pot synthesis with a 24 h reaction time.^40^ There remains, however, a desire rooted in the principles of green chemistry to develop a process, which can use raw biomass without, e.g., extracting glucose or employing mechanical pretreatment. Further, this should employ as few processing steps as possible and ideally synthesize the material in a one-pot process rather than via post-synthesis modification. The aim of the present work is to produce magnetic carbon composites with desirable properties for applications in adsorption and heterogeneous catalysis from whole AS in a one-pot synthesis process. The adsorption of model dye compounds is investigated as an environmental application, while catalytic studies focus on alkyne hydroalkoxylation—an important C–O bond-forming reaction. This work serves as an exemplar for a range of unprocessed waste lignocellulosic materials.

## 2 Results and Discussion

### 2.1 Physical and Chemical Characteristics of Fe/C

**Figure 1:**
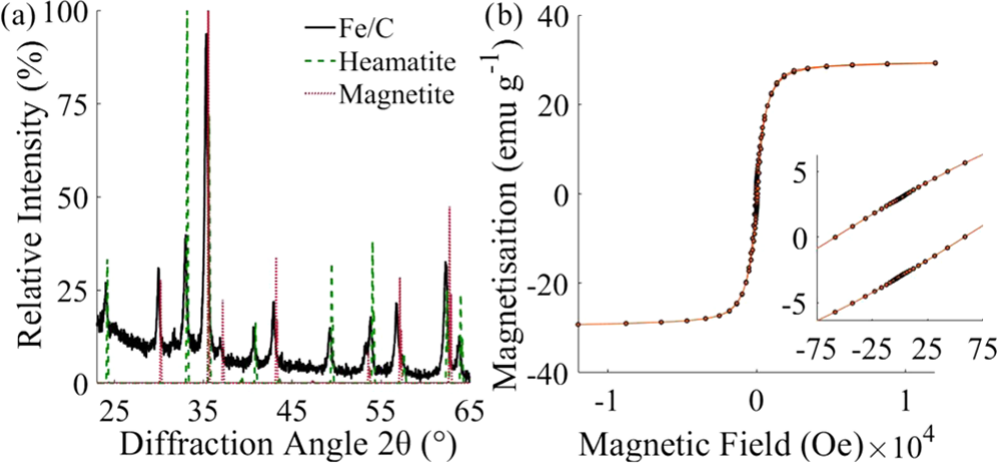
(a) XRD patterns for Fe/C particles compared to RRUFF spectroscopic references^41,42^ for magnetite and hematite between 17 and 65° obtained using a Cu tube with 1.54184 Å, scanning for 1 s at increments of 0.02°. (b) Magnetization hysteresis loop for Fe/C particles. The inset shows a small remnant of magnetization at zero field (∼10% of the total moment), measured using a Quantum Design SQUID-VSM Magnetometer MPMS3.

The powder X-ray diffractogram of the synthesized Fe/C is shown in Figure 1a. The diffractogram is dominated by the characteristic peaks of magnetite (Fe_3_O_4_) at 30.2, 35.6, 43.3, 57.2, and 62.8°; additionally, a smaller contribution from hematite (Fe_2_O_3_) is observed. Quantitative determination of the ratio of the two iron oxide phases was conducted using MATCH! Software (Crystal Impact GbR, Bonn, Germany). Fe/C comprised 73 ± 5.6% magnetite (Index# 96-151-3305^41^) and 27 ± 5.6% hematite (Index# 96-154-6384^42^). The predominance of magnetite is consistent with previous qualitative studies of magnetic iron–carbon composites synthesized from biomass.^40^ The sample was strongly attracted to an external magnet. Analysis of the sample employing a superconducting quantum interference device (SQUID) confirmed that the material was weakly ferromagnetic (Figure 1b). Based on previous studies, it is expected that only magnetite is precipitated from the precursor iron salts and sodium hydroxide during initial DAS soaking.^43,44^ Therefore, the presence of hematite implies that some oxidation of the formed iron oxide nanoparticles has occurred.^45^ The oxidation of magnetite under hydrothermal conditions has previously been observed by Li et al., with 41% of the formed magnetite in that system undergoing further oxidation at 275 °C under autogenous pressure.^46^

Analysis of the Fe/C composition by AAS indicated an iron content of 36.6 ± 5.4 wt %. This is consistent with the results of ICP-OES analysis, which indicate an iron content of 34.5 wt % based on the analysis of a single Fe/C sample. XRF results indicate an iron content of Fe/C of 37.5 ± 9.1 wt %, in agreement with both AAS and ICP-OES measurements.

XPS analysis of the surface composition of Fe/C reveals a surface iron content of 23.9 wt % (Table 1). As this is lower than the total iron content determined via bulk techniques, vide supra, it can be concluded that a significant proportion of the iron content resides within the bulk of the material as opposed to residing entirely in the surface region. Low concentrations of sodium, nitrogen, and sulfur were detected, presumably originating from raw AS. C 1s analysis revealed that the majority of the surface carbon is sp^3^ hybridized, some of which is oxygen functionalized; however, a smaller component of sp^2^ hybridized graphitic carbon is also present. No iron carbide was observed. XPS spectra are shown in Figures S1–S8.

**Table 1:** Surface Composition (wt %) Determined by Quantifying XPS Survey Scans Performed on Kratos Supra Instrument with a Monochromated Aluminum Source

analysis number	Na (wt %)	Fe (wt %)	O (wt %)	In (wt %)	N (wt %)	C (wt %)	S (wt %)
position 1 (20 eV pass)	1.1	22.8	21.9	3.4	2.9	47.1	0.8
position 2 (20 eV pass)	0.9	22.0	22.1	0.7	3.0	50.7	0.6
position 3 indium (20 eV pass)			17.1	67.7	0.5	14.7	
position 3 (10 eV pass)	1.2	27.3	22.6		2.8	45.4	0.8
position 4	1.3	21.0	22.2		2.9	52.1	0.4
position 5 (10 eV pass)	1.1	26.3	22.2		3.1	46.7	0.6

**Figure 2:**
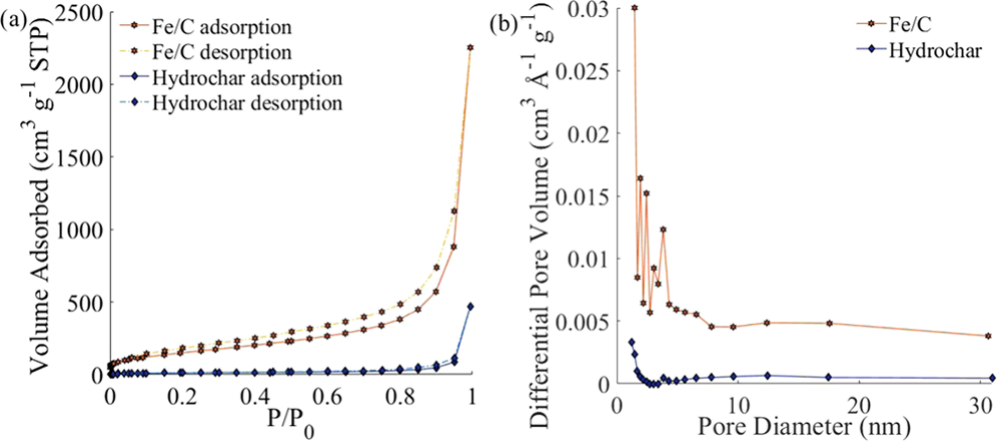
(a) N_2_ adsorption–desorption isotherm for F/C and hydrochar. (b) Pore size distribution for Fe/C and hydrochar. Data for both figures were obtained using a quadrasorb surface area analyzer and degasser.

BET analysis reveals surface areas of 546 and 194 m^2^g^–1^ for Fe/C and hydrochar, respectively. The surface area of Fe/C is significantly larger than the value of 272 m^2^ g^–1^ reported for a carbon-supported iron material prepared from lignin followed by wet impregnation with an iron salt, followed by post carbonization at 1100 °C.^47^ That Fe/C has a higher surface area than hydrochar is attributed to the action of iron oxide as a Lewis acid, facilitating the condensation of aromatic groups and the emission of volatile components. This increases the porosity and hence the surface area of this material.^48^ Because of this high surface area, it was possible to detect indium through the pores in a pressed sample via XPS (Table 1). Previously, surface areas of up to 1010 m^2^ g^–1^ have been obtained for hydrothermally synthesized iron-impregnated carbon.^49^ That work, however, employed a templating agent and thus requires additional preparation steps, higher processing temperature (800 °C), and the utilization of more costly and less environmentally friendly reactants. Nitrogen adsorption isotherms for both Fe/C and hydrochar are between Type II and IV (Figure 2). Both materials can therefore be classified as mesoporous and contain very few micropores.^50^ Similar behavior has been observed previously for carbon materials synthesized in hydrothermal environments in the presence of iron catalysts.^47,49^ Analysis of the pore size distribution shows that a majority of the pores are below 2 nm in diameter.

**Figure 3:**
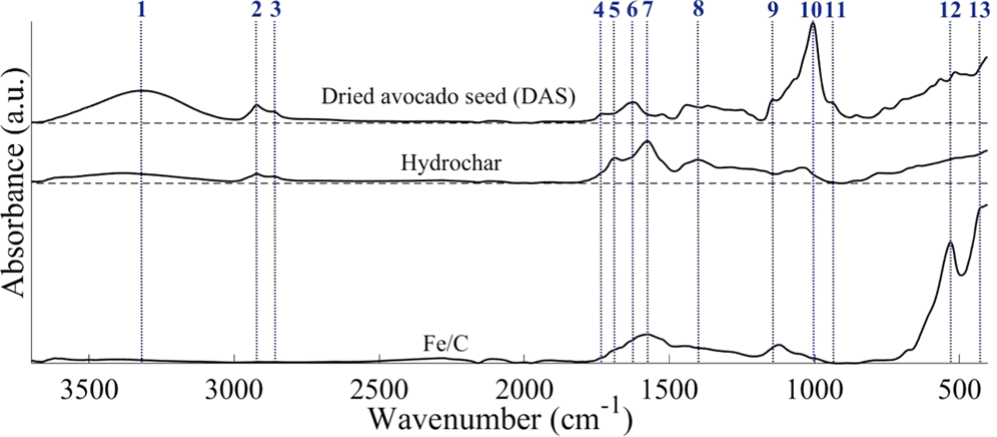
FT-IR spectra obtained for dried avocado seed (DAS), hydrochar, and Fe/C using a Shimadzu IRAffinity-1S spectrometer fitted with a Specac ATR plate. Analysis conditions: scans 4000, resolution 4 cm^–1^, absorbance mode. The numbered peaks are assigned as follows: 1: νO–H,; 2: ν_as_C–H; 3: ν_s_C–H; 4, 5 and 6: νC=O; 7: νC=C; 8: νC–C; 9: νC–O–C; 10: νC–O; 11: νC–C-O; 12 and 13: νFe–O.

The surface functionality of both Fe/C and the hydrochar synthesized in the absence of iron has been characterized by FT-IR spectroscopy (Figure 3). In addition, DAS has also been investigated for comparison. DAS exhibits strong absorbance in the O–H stretching region (**1**, 3650–3050 cm^–1^); however, this is significantly reduced in the hydrochar and fully eliminated in Fe/C. A similar trend is observed in the aliphatic region for asymmetric (**2**, 2919 cm^–1^) and symmetric (**3**, 2858 cm^–1^) C–H stretching, which is linked to the presence of methyl and methylene groups. The loss of aliphatic C–H bonds is attributed to condensation reactions occurring during HTC.^51^ These aldol polycondensation reactions are the final reactions within HTC that result in the formation of the extended polymeric structure of the synthesized carbonaceous material.^52^ That these are further reduced in Fe/C as compared to the hydrochar may indicate that the reaction has proceeded to a greater extent and that it may therefore be catalyzed by the presence of iron. Note also that the presence of iron oxide means that a lower fraction of the surface is hydrocarbonaceous when compared to hydrochar. Carbonyl (C=O) functionalities are also more apparent for DAS, as indicated by the presence of peaks associated with conjugated (**4**, 1737 cm^–1^) and unconjugated (**6**, 1619 cm^–1^) stretching. The large C=O stretching band (**5**, 1690 cm^–1^) present for hydrochar is attributed to conjugated aldehydes formed during HTC. FT-IR also provides evidence for the carbonization of the sample through the appearance of an aromatic C=C stretching band (**7**, 1575 cm^–1^) in the spectrum of Fe/C and hydrochar, in addition to C–C aromatic stretching (**8**, 1397 cm^–1^).^53,54^ This is supported by the loss of C–O–C (**9**, 1149 cm^–1^), C–O (**10**, 1005 cm^–1^), and C–C–O (**11**, 938 cm^–1^) stretches present in the DAS spectrum. These peaks are associated with ether C–O linkages from cellulose, lignin, and hemicelluloses, and elimination of this is indicative of the loss of this moiety during carbonization.^51^ The broad absorbance between 1475 and 1211 cm^–1^ present in the spectrum of DAS is attributed to a mixture of C–C aromatic, C–N, and C–O stretching vibrations. This is notably reduced in intensity for both hydrochar and Fe/C. Some surface C–O functionality does remain after HTC, as indicated by the peaks between 1050 and 1250 cm^–1^. Notably, hydrochar has relatively greater absorbance in this region than Fe/C. This is attributed not only to the increased carbonization noted by the reduction in aliphatic C–H vide supra but also to the likely displacement of these functionalities by the supported iron particles.

The FT-IR spectrum of Fe/C is dominated by Fe–O stretching associated with hematite (**12**, 532 cm^–1^, **13**, 440 cm^–1^).^55,56^ Magnetite typically exhibits a peak at 588 cm^–1^; if present, this is obscured in this work by the broad hematite peak. Bulk characterization (XRD, vide supra) indicated that magnetite was the dominant iron oxide phase present. ATR is a surface-sensitive technique, and therefore, the identification of hematite is indicative of the oxidation of the outer, air-exposed surface of the iron oxide.

The stability of the synthesized materials against the leaching of either metallic or organic components was qualitatively investigated by placing them in deionized water for 24 h and noting any discoloration. Previously synthesized iron–carbon composites which used sugarcane bagasse as the carbon source had shown discoloration of water after soaking.^40^ In the present study, hydrochar visibly discolored the solution to light gray immediately and to dark brown after 24 h. In contrast, the addition of Fe/C to deionized water resulted in no visible discoloration. The material leaching from the hydrochar is most likely unconverted hydrocarbonaceous material, with the brown coloration of the hydrochar indicative of the presence of tannins. Fe/C was obtained as a black powder, consistent with the higher degree of carbonization obtained in the presence of iron inferred from FT-IR spectra. This more carbonized material is, therefore, more resistant to leaching.

To quantify any reduction in iron content, and hence any leaching of iron, Fe/C was collected following soaking using an external magnet and analyzed using AAS, XRF, XRD, and FT-IR spectroscopy. All of the solid material was successfully collected with an external magnet. The recovered Fe/C was shown to have an iron loading of 35.6% by AAS and 36% by XRF, consistent with characterization of the as-synthesized material, indicating that negligible Fe had leached. XRD of Fe/C after leaching tests showed magnetite and hematite contents of 79.2% and 20.8%, respectively, consistent with the as-synthesized materials and therefore indicating that negligible oxidation or reduction of the oxide occurred.

### 2.2 Adsorption Testing of Fe/C

**Figure 4:**
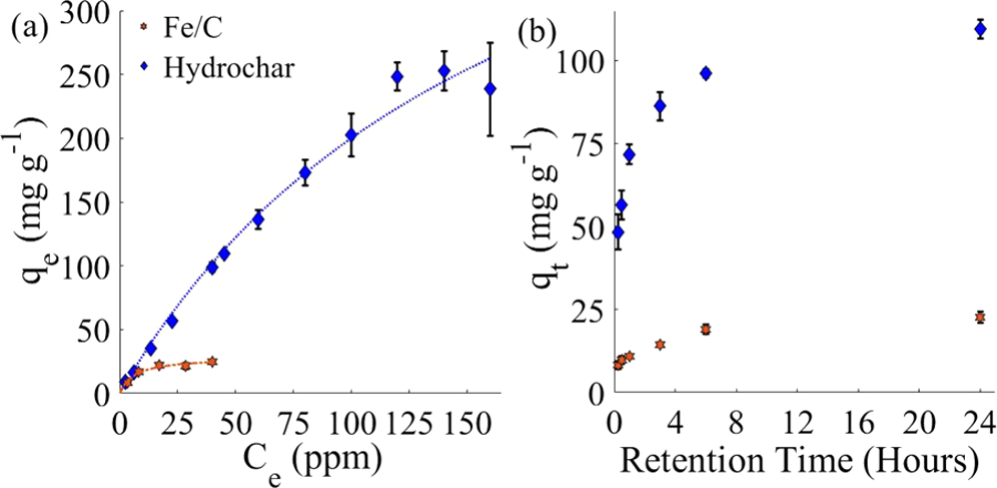
(a) Isothermal adsorption of methylene blue on Fe/C and hydrochar (0.01 g in 25 mL of solution) over 24 h at 30 °C, 180 rpm, fitted to Langmuir adsorption isotherms; (b) kinetic adsorption of methylene blue on Fe/C and hydrochar (0.01 g in 25 mL of solution), for 0.5, 1, 3, 6, and 24 h at 30 °C, 180 rpm. *C*_e_ is the concentration of the solution used, and *q*_e_ is the concentration of the solution at adsorption equilibrium.

The efficacy of the synthesized hydrochar and Fe/C have been tested in exemplar adsorption experiments. Synthetic dyes from the textile, paint, and printing industries are a major pollutant in effluent waste streams. Often these dyes are nonbiodegradable, toxic, and carcinogenic.^27,33^ These synthetic dyes consequently have adverse effects on the environment and human health. There is, therefore, a necessity to find methods of safe wastewater treatment for their removal. Of the available technologies, magnetic adsorbents have been increasingly proposed as a solution because of their simple operation, high separation, removal efficiency, and wide applicability.^34,57^ Methylene blue is widely used as an exemplar organic dye to test the efficiency of adsorbents.^58^ Kinetic and isothermal adsorption isotherms of methylene blue adsorption onto Fe/C and hydrochar synthesized in the present work are shown in Figure 4.

The isothermal adsorption data are fitted to a Langmuir isotherm model.^59^ Fe/C showed a maximum adsorption capacity of 24.8 ± 1.3 mg g^–1^. This adsorption capacity is consistent with previously studied Fe/C materials. For example, a graphene/magnetite material synthesized from high-value activated carbon exhibited a maximum adsorption capacity of 43.82 mg g^–1^.^58^ The results presented herein, therefore, demonstrate that materials produced from a low-value waste materials can exhibit performance competitive with such high-cost materials.

The synthesized hydrochar demonstrated an adsorption capacity of 246 ± 21 mg g^–1^. Carbonaceous materials (i.e., without iron) can exhibit a wide range of adsorption capacities; however, adsorbents produced from biomass typically have lower capacities than those produced conventionally, e.g., from activated carbon. For instance, adsorbents produced from olive stones, hazelnut shells, apricot stones, and walnut shells have been shown to have adsorption capacities of 22.1, 8.82, 4.11, and 3.53 mg g^–1^, respectively.^60^ The hydrochar obtained from AS herein shows significantly improved adsorption capacity when compared to these.

Hydrochar is clearly observed to be a more effective adsorbent than Fe/C for methylene blue both on a per mass and per surface area basis, having a surface area ∼2.8× smaller than that of Fe/C. This may be ascribed to its higher level of surface functionality, as shown by FT-IR spectroscopy (Section 2.1). Methylene blue binds to surface hydroxide and carbonyl groups on the carbon surface via hydrogen-bond interactions.^61^ It is however noteworthy that hydrochar exhibits leaching of organic components such as tannins in aqueous conditions. This may be undesirable where discoloration of the solution cannot be tolerated. Indeed, adsorbents are typically used in the tertiary stage of water treatment to remove any discoloration. The addition of Fe yields a material that is more resistant against leaching and therefore may be better suited to such applications despite its lower adsorption capacity. Furthermore, the removal of Fe/C from the solution after treatment is extremely facile, as this can be accomplished by magnetic separation.

**Figure 5:**
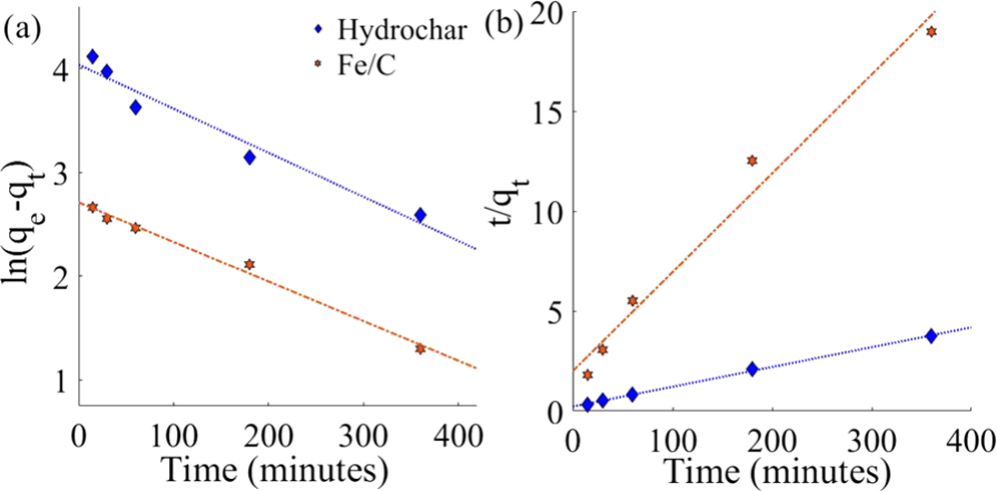
Pseudo-first (a) and -second (b) order fits to kinetic methylene blue adsorption data. Retention times of 0.25–24 h were used, with 16 and 45 ppm methylene blue solutions for Fe/C and hydrochar, respectively.

The results of kinetic studies of methylene blue adsorption on both Fe/C and hydrochar are shown in Figure 4b. After 6 h, both materials reach >80% of maximum adsorption. Fe/C shows similar kinetics to previously reported Fe/C materials synthesized by conventional methods, e.g., 54% of maximum adsorption is achieved after 2.5 h.^40^ Kinetic adsorption data were fitted to Lagergren pseudo-first-order and pseudo-second-order rate equations (eqs 1 and 2).^62^
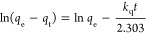
1
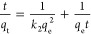
2The fitted data are shown in Figure 5 and the extracted adsorption capacity at equilibrium and rate constant are shown in Table 2 alongside *R*^2^ values. It is apparent that pseudo-second-order rate equations describe the adsorption process well and significantly better than pseudo-first-order kinetics in the case of hydrochar. Adsorption is therefore inferred to follow second-order kinetics. Mechanistically, this suggests that methylene blue is chemisorbed onto the hydrochar surface (rather than being physisorbed).^63^ This results in improved retention and hence better performance in adsorption applications.

**Table 2:** Extracted Data from Pseudo- First and -Second-Order Fitting of Experimental Kinetic Adsorption Data for Methylene Blue Over Fe/C and Hydrochar

		pseudo-first order			pseudo-second order		
sample	exp *q*_e_ (mg g^–1^)	*q*_e_ cal (mg g^–1^)	*k*_1_ (min^–1^)	*R*^2^	*q*_e_ cal (mg g^–1^)	*k*_1_ (mg g^–1^ min^–1^)	*R*^2^
Fe/C	24.8 ± 1.3	14.9	0.0088	0.9907	20.4	0.0012	0.9777
hydrochar	246 ± 21	54.6	0.0097	0.9553	101	0.00043	0.9985

**Figure 6:**
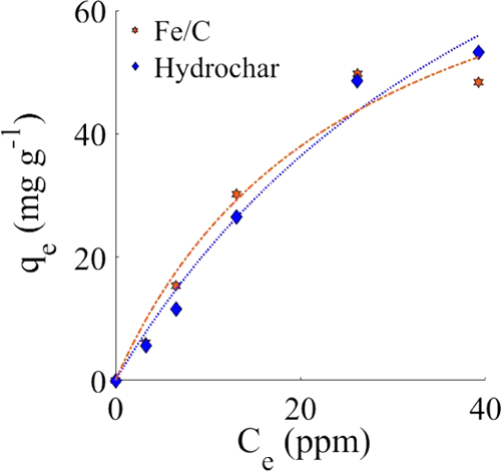
Isothermal adsorption of indigo carmine on Fe/C and hydrochar (0.01 g in 25 mL of solution) over 24 h at 30 °C, 180 rpm. Langmuir adsorption isotherms fits are shown. *C*_e_ is the concentration of solution used, and *q*_e_ is the concentration of the solution at adsorption equilibrium.

Electronic interactions play a key role in adsorption processes: an increase in adsorption is expected if the adsorbate and adsorbent have opposing charges, causing positive interactions.^64^ Therefore, it is speculated that the surface charge of Fe/C is positive, thereby lowering its adsorption capacity for the positively charged cationic dye methylene blue relative to that of hydrochar.^65^ Adsorption of an alternative adsorbate—indigo carmine, which is an anionic dye (negatively charged)—was therefore investigated (Figure 6).^66^ The maximum adsorption capacities of indigo carmine on Fe/C and hydrochar particles were determined to be 49.0 ± 3.7 and 50.9 ± 2.9 mg g^–1^, respectively. Both the hydrochar and Fe/C therefore show very similar adsorption properties. Fe/C, however, has the advantage of facile magnetic separation from solution. These results support the hypothesis that the comparatively lower maximum adsorption capacity of Fe/C for methylene blue is caused by electronic repulsion effects. The affinity of the adsorbents for indigo carmine may be further enhanced by the presence of graphitic carbon on the hydrochar as revealed by XPS (Section 2.1). Interaction between delocalized electrons in the indigo carmine and delocalized electrons in the adsorbate will facilitate adsorption via π-interactions.^67^ It should also be noted that, typically, the maximum adsorption capacity for indigo carmine is generally lower than methylene blue, e.g., a commercial activated carbon has exhibited a maximum adsorption capacity 7 times lower for indigo carmine (135 mg g^–1^) than for methylene blue.^68^

### 2.3 Catalytic Testing of Fe/C

The catalytic efficiency of Fe/C toward the hydroalkoxylation of phenylacetylene with ethylene glycol was evaluated and compared to both hydrochar and unsupported magnetite nanoparticles. This reaction offers an atom-economical method of creating a carbonyl bond using an alkene or alkyne and an alcohol.^69^ The reaction was first demonstrated in 1936 using a mercuric oxide catalyst with boron trifluoride-based co-catalysts.^70^ Since then, systems based on Hg, Os, Ru, Pt, Ag, and Au within organic complexes and as oxides have been widely reported.^71^ Many of these materials, however, suffer from challenges around high toxicity or high cost. There has therefore been interest in the application of iron salts to overcome these challenges.^72−74^ The first studies on heterogeneous iron catalysts were presented in 2015 by Antoniotti et al.^75^ These employed montmorillonite-supported iron, achieving yields of up to 86% for the hydroalkoxylation of 5-methyl-2,2-diphenyl-4-hexen-1-ol to 2,2-dimethyl-5,5-diphenyltetrahydro-2H-pyran in dimethyl carbonate (DMC) as a solvent. The further development of low-cost, active, heterogeneous catalysts based on earth-abundant materials remains a desirable objective.

Each catalytic test was performed in triplicate, and results were averaged. Unsupported magnetite nanoparticles were found to successfully yield 2B13D with a yield of 22.7%. This serves as a benchmark against which to compare the catalytic efficiency of the HTC-synthesized materials. Fe/C showed a significantly greater product yield of 45%. The same total quantity of iron oxide is present in reactions involving magnetite nanoparticles and those employing Fe/C. Therefore, this difference is reflective of the higher activity of the HTC-derived material and its differing physicochemical characteristics, including oxidation state, available surface area, *etc.* Previously, the hydroalkoxylation of phenylacetylene with ethylene glycol has been studied utilizing a homogeneous Au(I)/AgBF_4_ catalyst.^76^ In contrast to the present work, Au(I)/AgBF_4_ demonstrated high selectivity for the alternative regioisomer, producing 2-methyl-2-phenyl-1,3-dioxolane (2P13D) with a yield of 87% (corresponding to a selectivity of 75%). No 2P13D is observed over Fe/C. The exceptional regioselectivity for 2B13D observed over Fe/C is attributed to the conformation adopted by the transition state, with bonding to heterogeneous iron active site taking place via the enol functionality.^77^ High selectivity, and the ability to produce exclusively one isomer, is a key objective in catalyst development and one successfully demonstrated by the material synthesized in this work. Hydrochar presented a lower product yield than either iron-based material at 11.2%. While the lower surface area of hydrochar cf. Fe/C contributes to this, the presence of iron in Fe/C enhances catalytic activity via electrophilic bonding to the alkyne.^78^ There is then likely a synergistic interaction between iron and carbon, whereby the hydrochar facilitates hydroalkoxylation through stabilizing the hydroxide group, resulting in the formation of the stabilized enol transition state.^78,79^ These studies demonstrate the potential to synthesize active and selective catalytic materials from low-value waste and co-product streams via HTC.

## 3 Conclusions

A facile, rapid (3 h), and relatively low temperature (230 °C) one-pot HTC synthesis procedure has been demonstrated to synthesize a carbon-supported metal oxide material, with demonstrated applications in adsorption for environmental applications and as a safe and sustainable heterogeneous catalyst. Avocado seeds, a waste material with an expanding production, have been used an exemplar carbon source for the first time. The methodology presented can however be applied to a wide range of lignocellulosic waste streams. The synthesized Fe/C consists predominately of magnetite, allowing easy magnetic separation after application. When employed as a sorbent, Fe/C showed the potential to be used as a selective adsorbent for anionic adsorbates in waste streams with a maximum adsorption capacity for indigo carmine of ∼49 mg g^–1^. Additionally, Fe/C showed enhanced catalytic efficiency when compared to magnetite nanoparticles, exhibiting a 2-benzyl-1,3-dioxolane yield in the hydroalkoxylation of phenylacetylene of ∼45%, versus ∼23% for the nanoparticles. Additionally, exceptional regioselectivity toward 2-benzyl-1,3-dioxolane was observed. These results suggest a role for HTC in the production of carbon materials for high-value environmental and other applications, rooted in the principles of the circular economy.

## 4 Experimental Section

### 4.1 Materials

Iron (III) nitrate nonahydrate (Fe(NO_3_)_3_·9H_2_O), iron (II) sulfate heptahydrate (FeSO_4_·7H_2_O), acetone, hexane, nitric acid (64–66%, HNO_3_), sodium hydroxide (NaOH), iodobenzene, phenylacetylene, potassium carbonate, indigo carmine, and ethylene glycol were all purchased from Sigma-Aldrich (Dorset, U.K.). Methylene blue trihydrate was purchased from Bio Basic (Cambridgeshire, U.K.). Deionized water was obtained from a Suez L300130 (>1 MΩ cm). Carbon dioxide (N5.0, BOC) and helium (A Grade, 99.996%, BOC) were supplied by BOC. Iron (II, III) oxide (97%, metals basis) was purchased from Alfa Aesar (Lancashire, U.K.). All materials were used as received unless otherwise stated.

### 4.2 Synthesis Procedure

Magnetic carbon composites (Fe/C) were synthesized by the following procedure. AS were dried in an oven (Memmert, Schwabach, Germany) at 100 °C for 24 h and then subsequently stored in a cold room at 5 °C. Dried avocado seeds (DAS) were pretreated under reflux in sodium hydroxide (0.1 M, 50 mL) at 70 °C for 2 h to remove tannins and dyes prior to separation by filtration and washing with deionized water (3 × 50 mL).^80^ The seeds were then dried in an oven (100 °C, 24 h). DAS were soaked in a 1:2 mole ratio Fe^2+^/Fe^3+^ solution (0.5 M FeSO_4_·7H_2_O, 1 M Fe(NO_3_)_3_·9H_2_O, 50 mL) for ≥3 days. Subsequently, the soaked AS and the iron solution were transferred to a 300 mL autoclave (Series 3050, Parr instrument company, IL) to which NaOH solution (1 M, 50 mL) was added. The vessel was pressurized with CO_2_ (30 bar) and transferred to an aluminum block, which had been preheated to 230 °C using a heating mantle (C-MAG HS7, IKA, Oxford, U.K.). The temperature was monitored using a thermocouple. An internal temperature of 230 °C was achieved after 45 min, and this temperature was maintained for 3 h. The reaction was then quenched in a water bath for 30 min. The solid powder was collected by vacuum filtration and soaked in acetone (50 mL) for 24 h. It was then subjected to a further vacuum filtration and then washed with deionized water (3 × 50 mL) and dried in an oven (100 °C, 24 h). The Fe/C was then heated in a tube furnace (Three Zone Horizontal, Elite, Leicestershire, U.K.) to 400 °C (ramp rate 6.5 °C min^–1^) under constant nitrogen gas flow and held at that temperature for 1 h before cooling to room temperature. The resultant product was ground using a pestle and mortar and sieved to a size fraction of 38–212 μm using mesh steel sieves. Iron-free hydrochar (hereafter referred to as hydrochar) was also synthesized following the same procedure as above but omitting the iron soaking procedure and adding an additional 50 mL of deionized water to the autoclave during carbonization to ensure that the same liquid volume was used in both cases. The overall yield of carbonaceous material, based on the initial mass of AS weight, was 18.2 ± 4.9%. Negligible losses of iron were observed with near-full iron incorporation into the final Fe/C.

Magnetite nanoparticles were synthesized via a conventional route, as described by Liu et al.,^81^ to provide a reference against which to measure the catalytic activity of the HTC-derived materials (Section 4.5). FeCl_3_·6H_2_O (6.1 g, 22.6 mmol) and FeSO_4_·7H_2_O (4.2 g, 15.1 mmol) were dissolved in deionized water (100 mL) and heated to 90 °C. NH_4_OH (10 mL, 25%) was then added under stirring. After 30 min, the black precipitate was collected by vacuum filtration and dried in an oven (Memmert, Schwabach, Germany) (100 °C, 24 h). The product magnetite nanoparticles were used in the hydroalkoxylation reaction directly.

### 4.3 Fe/C Characterization

The crystal structure of the synthesized Fe/C was analyzed using powder X-ray diffraction (XRD). XRD was conducted on a D2 Phaser (Bruker, MA) using a Cu tube (λ = 1.54184 Å) with 1 s scanning increments of 0.02° between 24 and 65°. Compositional analysis was conducted by atomic absorption spectroscopy (AAS), X-ray fluorescence (XRF), and inductively coupled plasma-optical emission spectrometry (ICP-OES). Surface area and pore size analyses were performed using a quadrasorb surface area analyzer and degasser (Micromeritics, Norcross) following Brunauer–Emmett–Teller (BET) theory. For AAS, samples were digested in 1:3 nitric:hydrochloric acid stock solution (aqua regia) and diluted to 1–10 ppm solutions in 1% nitric acid prior to analysis using a AAS AAnalyst 400 (PerkinElmer, MA). The iron powder was digested and analyzed following the same procedure to produce standards for calibration.^82^ XRF was conducted on an Olympus Delta DP-2000-C (GP Technical Equipment, Savannah) using a titanium filter, tube voltage of 50 kV and a current of 55 μA. For ICP-OES, Fe/C was dissolved using a 1:1 mixture of nitric and perchloric acid and then analyzed using a Spectro Ciros Vision ICP-OES (AMETEK Materials, Kleve Germany). Further surface composition was examined using X-ray photoelectron spectroscopy (XPS) performed on Kratos Supra (Kratos Analytical Ltd., Manchester, U.K.) instrument with a monochromated aluminum source at two analyses per sample, each of area 700 μm × 300 μm. Survey scans were collected between 1200 and 0 eV binding energy, at 160 eV pass energy, 1 eV intervals, and 300 s/sweep, with one sweep being collected. High-resolution Fe 2p, O 1s, C 1s, N 1s, and In 3d were also collected. The data was analyzed using CasaXPS software. Characterization of surface functionalities was conducted by Fourier-transform infrared (FT-IR) spectroscopy performed on a IRAffinity-1S (Shimadzu, Buckinghamshire, U.K.) at a resolution of 4 cm^–1^ with 4000 scans over the range 400–4000 cm^–1^. FT-IR data was normalized using an adaptive baseline (25% coarseness) using optical spectroscopy software (Spectragryph, http://spectroscopy.ninja, Germany). Magnetization was measured using a Quantum Design (London, U.K.) superconducting quantum interference device (SQUID)-vibrating sample magnetometer (VSM) MPMS3.

### 4.4 Adsorption Studies

The isothermal adsorption capacity and the kinetics of adsorption were investigated. All uptake studies were conducted in an Infors HT multitron shaker at 30 °C and 180 rpm. Isothermal experiments were performed over 24 h using 0.01 g of adsorbent and 25 mL of 4–40 ppm methylene blue solution for Fe/C and 25 mL of 4–160 ppm methylene blue solution for hydrochar. Kinetic studies employed retention times of 0.25–24 h with 16 and 45 ppm methylene blue solutions for Fe/C and hydrochar, respectively. After the specified retention time, Fe/C was magnetically separated; alternatively, for nonmagnetic hydrochar, separation was conducted by filtration. The remaining solutions were analyzed using a UV–vis spectrophotometer (Genesys 150, ThermoFisher Scientific, MA) (λ = 634 nm). The quantity of methylene blue adsorbed, *q*_e_ (mg/g), and *q*_t_ (mg/g) were calculated, as shown in eqs 3 and 4
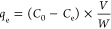
3
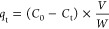
4where *C*_0_ and *C*_e_ are initial and equilibrium concentrations of methylene blue ppm (mg L^–1^), *V* is the volume of solution (L), and *W* is the weight of the adsorbent used (g).

Isothermal adsorption studies were also performed on Fe/C and hydrochar using indigo carmine as an alternative adsorbate. This was performed by the same analysis procedure with concentrations between 3 and 36 ppm over a time of 24 h and analyzed using UV–vis spectrophotometry (λ = 610 nm).

### 4.5 Catalytic Testing

**Scheme 1:**
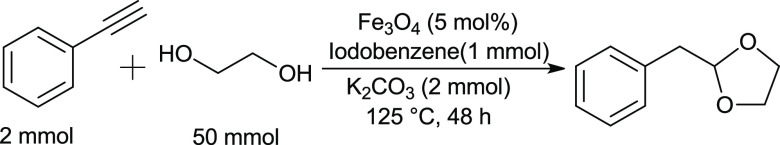
Schematic of the Hydroalkoxylation of Phenylacetylene With Ethylene Glycol to Yield 2-Benzyl-1,3-Dioxolane

The catalytic performance of Fe/C was evaluated in the hydroalkoxylation of phenylacetylene with ethylene glycol, yielding 2-benzyl-1,3-dioxolane (2B13D) as the desired product (Scheme 1).^69,70^ Iodobenzene (1 mmol, 0.20 g/0.11 mL), phenylacetylene (2 mmol, 0.20 g/0.22 mL), potassium carbonate (2 mmol, 0.276 g), and the catalyst (0.058 g magnetite nanoparticles, 0.116 g Fe/C or 0.116 g hydrochar) were dissolved in ethylene glycol (3.34 g/3 mL) and stirred for 48 h under reflux. The quantities of magnetite nanoparticles and Fe/C were selected to give the same quantity of iron oxide (∼0.25 mmol) in both cases. After stirring, the solid material was separated using an external magnet or via filtration in the case of hydrochar. Deionized water (5 mL) and hexane (5 mL) were then added to the remaining solution, and the hexane layer was separated. The hexane extract was analyzed by GC-MS (Shimadzu QP2010 SE, Buckinghamshire, U.K.) equipped with a DB5-MS column (24.5 m, 0.25 mm, 0.25 μm). Briefly, 10 μL of hexane was injected at 250 °C with an initial oven temperature of 40 °C, which was raised to 240 °C at 10 °C/min. High purity helium (A Grade, 99.996%, BOC) was used as a carrier gas at a flow rate of 1 mL/min. The product concentration was quantified using external calibration standards with an *R*^2^ value of 0.991. Where possible, all results are reported with 95% confidence intervals from five separately synthesized Fe/C samples and three separately synthesized hydrochar samples.

## Supporting Information Available

Figures S1–S8: high-resolution XPS spectra for Fe/C focused on Fe 2p and C 1s scans in different positions on the sample (PDF)

## References

[ref1] Arancon R. A. D.; Lin C. S. K.; Chan K. M.; Kwan T. H.; Luque R. Advances on Waste Valorization: New Horizons for a More Sustainable Society. Energy Sci. Eng. 2013, 1, 53–71. 10.1002/ese3.9.

[ref2] Karak T.; Bhagat R. M.; Bhattacharyya P. Municipal Solid Waste Generation, Composition, and Management: The World Scenario. Crit. Rev. Environ. Sci. Technol. 2012, 42, 1509–1630. 10.1080/10643389.2011.569871.

[ref3] Nayak A.; Bhushan B. An Overview of the Recent Trends on the Waste Valorization Techniques for Food Wastes. J. Environ. Manage. 2019, 233, 352–370. 10.1016/j.jenvman.2018.12.041. 30590265

[ref4] Amulya K.; Dahiya S.; Venkata Mohan S. Building a Bio-Based Economy through Waste Remediation: Innovation towards Sustainable Future; Elsevier Inc., 2016.

[ref5] Smith A. M.; Ross A. B. Production of Bio-Coal, Bio-Methane and Fertilizer from Seaweed via Hydrothermal Carbonisation. Algal Res. 2016, 16, 1–11. 10.1016/j.algal.2016.02.026.

[ref6] Smith A. M.; Whittaker C.; Shield I.; Ross A. B. The Potential for Production of High Quality Bio-Coal from Early Harvested Miscanthus by Hydrothermal Carbonisation. Fuel 2018, 220, 546–557. 10.1016/j.fuel.2018.01.143.

[ref7] Mäkelä M.; Benavente V.; Fullana A. Hydrothermal Carbonization of Lignocellulosic Biomass: Effect of Process Conditions on Hydrochar Properties. Appl. Energy 2015, 155, 576–584. 10.1016/j.apenergy.2015.06.022.

[ref8] Funke A.; Ziegler F. Hydrothermal Carbonization of Biomass: A Summary and Discussion of Chemical Mechanisms for Process Engineering. Biofuels, Bioprod. Biorefin. 2010, 6, 160–177. 10.1002/bbb.198.

[ref9] Hoekman S. K.; Broch A.; Robbins C. Hydrothermal Carbonization (HTC) of Lignocellulosic Biomass. Energy Fuels 2011, 25, 1802–1810. 10.1021/ef101745n.

[ref10] Atiqah Nasir N.; Davies G.; McGregor J. Tailoring Product Characteristics in the Carbonisation of Brewers’ Spent Grain through Solvent Selection. Food Bioprod. Process. 2020, 120, 41–47. 10.1016/j.fbp.2019.12.010.

[ref11] Davies G.; El Sheikh A.; Collett C.; Yakub I.; McGregor J. In Catalytic Carbon Materials from Biomass; Sadjadi S. B., Ed.; Elsevier, 2021, Chapter 5; pp 161–195.

[ref12] He C.; Giannis A.; Wang J. Y. Conversion of Sewage Sludge to Clean Solid Fuel Using Hydrothermal Carbonization: Hydrochar Fuel Characteristics and Combustion Behavior. Appl. Energy 2013, 111, 257–266. 10.1016/j.apenergy.2013.04.084.

[ref13] Yao Z.; Ma X. Characteristics of Co-Hydrothermal Carbonization on Polyvinyl Chloride Wastes with Bamboo. Bioresour. Technol. 2018, 247, 302–309. 10.1016/j.biortech.2017.09.098. 28950139

[ref14] Haque N.; Somerville M. Techno-Economic and Environmental Evaluation of Biomass Dryer. Procedia Eng. 2013, 56, 650–655. 10.1016/j.proeng.2013.03.173.

[ref15] Reza M. T.; Yan W.; Uddin M. H.; Lynam J. G.; Hoekman S. K.; Coronella C. J.; Vásquez V. R. Reaction Kinetics of Hydrothermal Carbonization of Loblolly Pine. Bioresour. Technol. 2013, 139, 161–169. 10.1016/j.biortech.2013.04.028. 23651600

[ref16] Cai J.; Li B.; Chen C.; Wang J.; Zhao M.; Zhang K. Hydrothermal Carbonization of Tobacco Stalk for Fuel Application. Bioresour. Technol. 2016, 220, 305–311. 10.1016/j.biortech.2016.08.098. 27589825

[ref17] Zhang L.; Wang Q.; Wang B.; Yang G.; Lucia L. A.; Chen J. Hydrothermal Carbonization of Corncob Residues for Hydrochar Production. Energy Fuels 2015, 29, 872–876. 10.1021/ef502462p.

[ref18] Islam M. A.; Ahmed M. J.; Khanday W. A.; Asif M.; Hameed B. H. Mesoporous Activated Coconut Shell-Derived Hydrochar Prepared via Hydrothermal. J. Environ. Manage. 2017, 203, 237–244. 10.1016/j.jenvman.2017.07.029. 28783020

[ref19] White R. J.; Yoshizawa N.; Antonietti M.; Titirici M.-M. A Sustainable Synthesis of Nitrogen-Doped Carbon Aerogels. Green Chem. 2011, 13, 2428 10.1039/c1gc15349h.

[ref20] Wu Q.; Li W.; Liu S.; Jin C. Hydrothermal Synthesis of N-Doped Spherical Carbon from Carboxymethylcellulose for CO_2_ capture. Appl. Surf. Sci. 2016, 369, 101–107. 10.1016/j.apsusc.2016.02.022.

[ref21] Hu B.; Wang K.; Wu L.; Yu S. H.; Antonietti M.; Titirici M. M. Engineering Carbon Materials from the Hydrothermal Carbonization Process of Biomass. Adv. Mater. 2010, 22, 813–828. 10.1002/adma.200902812. 20217791

[ref22] Tran H. N.; Huang F. C.; Lee C. K.; Chao H. P. Activated Carbon Derived from Spherical Hydrochar Functionalized with Triethylenetetramine: Synthesis, Characterizations, and Adsorption Application. Green Process. Synth. 2017, 6, 565–576. 10.1515/gps-2016-0178.

[ref23] Gai C.; Zhang F.; Yang T.; Liu Z.; Jiao W.; Peng N.; Liu T.; Lang Q.; Xia Y. Hydrochar Supported Bimetallic Ni-Fe Nanocatalysts with Tailored Composition, Size and Shape for Improved Biomass Steam Reforming Performance. Green Chem. 2018, 20, 2788–2800. 10.1039/C8GC00433A.

[ref24] Titirici M. M.; White R. J.; Brun N.; Budarin V. L.; Su D. S.; Del Monte F.; Clark J. H.; MacLachlan M. J. Sustainable Carbon Materials. Chem. Soc. Rev. 2015, 44, 250–290. 10.1039/C4CS00232F. 25301517

[ref25] Sánchez F.; Araus K.; Domínguez M. P.; Miguel G. S. Thermochemical Transformation of Residual Avocado Seeds: Torrefaction and Carbonization. Waste Biomass Valoriz. 2017, 8, 2495–2510. 10.1007/s12649-016-9699-6.

[ref26] Xue J.; Chellappa T.; Ceylan S.; Goldfarb J. L. Enhancing Biomass + Coal Co-Firing Scenarios via Biomass Torrefaction and Carbonization: Case Study of Avocado Pit Biomass and Illinois No. 6 Coal. Renewable Energy 2018, 122, 152–162. 10.1016/j.renene.2018.01.066.

[ref27] Palma C.; Lloret L.; Puen A.; Tobar M.; Contreras E. Production of Carbonaceous Material from Avocado Peel for Its Application as Alternative Adsorbent for Dyes Removal. Chin. J. Chem. Eng. 2016, 24, 521–528. 10.1016/j.cjche.2015.11.029.

[ref28] OECD-FAO Agricultural Outlook 2021–2030. Food and Agriculture Organization: United Nations, 2020.

[ref29] Lin Y.; Ma X.; Peng X.; Yu Z.; Fang S.; Lin Y.; Fan Y. Combustion, Pyrolysis and Char CO2-Gasification Characteristics of Hydrothermal Carbonization Solid Fuel from Municipal Solid Wastes. Fuel 2016, 181, 905–915. 10.1016/j.fuel.2016.05.031.

[ref30] Durak H.; Aysu T. Effect of Pyrolysis Temperature and Catalyst on Production of Bio-Oil and Bio-Char from Avocado Seeds. Res. Chem. Intermed. 2015, 41, 8067–8097. 10.1007/s11164-014-1878-0.

[ref31] Michelin M.; Ruiz H. A.; Silva D. P.; Ruzene D. S.; Teixeira J. A.; Polizeli M. L. T. M. Cellulose from Lignocellulosic Waste BT - Polysaccharides: Bioactivity and Biotechnology, Ramawat K. G., Mérillon J.-M., Eds.; Springer International Publishing: Cham, 2025; pp 475−511.

[ref32] Abiemwense G. Influence of Hydrothermal Treatment Duration on the Nutritional Quality of Avocado Pear (*Persia americana*) Seed Meal for Livestock Feeding. Anim. Res. Int. 2017, 14, 2759–2763.

[ref33] Xu P.; Zeng G. M.; Huang D. L.; Feng C. L.; Hu S.; Zhao M. H.; Lai C.; Wei Z.; Huang C.; Xie G. X.; et al. Use of Iron Oxide Nanomaterials in Wastewater Treatment: A Review. Sci. Total Environ. 2012, 424, 1–10. 10.1016/j.scitotenv.2012.02.023. 22391097

[ref34] Reddy L. H.; Arias J. L.; Nicolas J.; Couvreur P. Magnetic Nanoparticles: Design and Characterization, Toxicity and Biocompatibility, Pharmaceutical and Biomedical Applications. Chem. Rev. 2012, 112, 5818–5878. 10.1021/cr300068p. 23043508

[ref35] Sun H.; Zhou G.; Liu S.; Ang H. M.; Tadé M. O.; Wang S. Nano-Fe0 Encapsulated in Microcarbon Spheres: Synthesis, Characterization, and Environmental Applications. ACS Appl. Mater. Interfaces 2012, 4, 6235–6241. 10.1021/am301829u. 23101516

[ref36] Ma Q.; Cui L.; Zhou S.; Li Y.; Shi W.; Ai S. Iron Nanoparticles in Situ Encapsulated in Lignin-Derived Hydrochar as an Effective Catalyst for Phenol Removal. Environ. Sci. Pollut. Res. 2018, 25, 20833–20840. 10.1007/s11356-018-2285-7. 29761356

[ref37] Zhu X.; Qian F.; Liu Y.; Matera D.; Wu G.; Zhang S.; Chen J. Controllable Synthesis of Magnetic Carbon Composites with High Porosity and Strong Acid Resistance from Hydrochar for Efficient Removal of Organic Pollutants: An Overlooked Influence. Carbon 2016, 99, 338–347. 10.1016/j.carbon.2015.12.044.

[ref38] Liu Y.; Zhu X.; Qian F.; Zhang S.; Chen J. Magnetic Activated Carbon Prepared from Rice Straw-Derived Hydrochar for Triclosan Removal. RSC Adv. 2014, 4, 63620–63626. 10.1039/C4RA11815D.

[ref39] Altay B. N.; Aksoy B.; Banerjee D.; Maddipatla D.; Fleming P. D.; Bolduc M.; Cloutier S. G.; Atashbar M. Z.; Gupta R. B.; Demir M. Lignin-Derived Carbon-Coated Functional Paper for Printed Electronics. ACS Appl. Electron. Mater. 2021, 3, 3904–3914. 10.1021/acsaelm.1c00502.

[ref40] Rattanachueskul N.; Saning A.; Kaowphong S.; Chumha N.; Chuenchom L. Magnetic Carbon Composites with a Hierarchical Structure for Adsorption of Tetracycline, Prepared from Sugarcane Bagasse via Hydrothermal Carbonization Coupled with Simple Heat Treatment Process. Bioresour. Technol. 2017, 226, 164–172. 10.1016/j.biortech.2016.12.024. 28006734

[ref41] Lafuente B.; Downs R. T.; Yang H.; Stone N. Software for Identification and Refinement of Cell Parameters from Powder Diffraction Data of Minerals Using the RRUFF Project and American Mineralogist Crystal Structure Databases. Magnetite R061111, https://rruff.info/i/R061111 (accessed Sep 2, 2019).

[ref42] Lafuente B.; Downs R. T.; Yang H.; Stone N. Software for Identification and Refinement of Cell Parameters from Powder Diffraction Data of Minerals Using the RRUFF Project and American Mineralogist Crystal Structure Databases. Hematite R110013, https://rruff.info/hematite/R110013 (accessed Sep 2, 2019).

[ref43] Khalil M. I. Co-Precipitation in Aqueous Solution Synthesis of Magnetite Nanoparticles Using Iron(III) Salts as Precursors. Arab. J. Chem. 2015, 8, 279–284. 10.1016/j.arabjc.2015.02.008.

[ref44] Yu W.; Zhang T.; Zhang J.; Qiao X.; Yang L.; Liu Y. The Synthesis of Octahedral Nanoparticles of Magnetite. Mater. Lett. 2006, 60, 2998–3001. 10.1016/j.matlet.2006.02.032.

[ref45] Šutka A.; Lagzdina S.; Juhnevica I.; Jakovlevs D.; Maiorov M. Precipitation Synthesis of Magnetite Fe3O4 Nanoflakes. Ceram. Int. 2014, 40, 11437–11440. 10.1016/j.ceramint.2014.03.140.

[ref46] Li Z.; Chanéac C.; Berger G.; Delaunay S.; Graff A.; Lefèvre G. Mechanism and Kinetics of Magnetite Oxidation under Hydrothermal Conditions. RSC Adv. 2019, 9, 33633–33642. 10.1039/C9RA03234G. PMC9073545 35528876

[ref47] Demir M.; Kahveci Z.; Aksoy B.; Palapati N. K. R.; Subramanian A.; Cullinan H. T.; El-Kaderi H. M.; Harris C. T.; Gupta R. B. Graphitic Biocarbon from Metal-Catalyzed Hydrothermal Carbonization of Lignin. Ind. Eng. Chem. Res. 2015, 54, 10731–10739. 10.1021/acs.iecr.5b02614.

[ref48] Zhu X.; Liu Y.; Qian F.; Zhou C.; Zhang S.; Chen J. Role of Hydrochar Properties on the Porosity of Hydrochar-Based Porous Carbon for Their Sustainable Application. ACS Sustainable Chem. Eng. 2015, 3, 833–840. 10.1021/acssuschemeng.5b00153.

[ref49] Sevilla M.; Fuertes A. B. Catalytic Graphitization of Templated Mesoporous Carbons. Carbon 2006, 44, 468–474. 10.1016/j.carbon.2005.08.019.

[ref50] Donohue M. D.; Aranovich G. L. Classification of Gibbs Adsorption Isotherms. Adv. Colloid Interface Sci. 1998, 76–77, 137–152. 10.1016/S0001-8686(98)00044-X.

[ref51] Kang S.; Li X.; Fan J.; Chang J. Characterization of Hydrochars Produced by Hydrothermal Carbonization of Lignin, Cellulose, d-Xylose, and Wood Meal. Ind. Eng. Chem. Res. 2012, 51, 9023–9031. 10.1021/ie300565d.

[ref52] Higgins L. J. R.; Brown A. P.; Harrington J. P.; Ross A. B.; Kaulich B.; Mishra B. Evidence for a Core-Shell Structure of Hydrothermal Carbon. Carbon 2020, 161, 423–431. 10.1016/j.carbon.2020.01.060.

[ref53] Parshetti G. K.; Kent Hoekman S.; Balasubramanian R. Chemical, Structural and Combustion Characteristics of Carbonaceous Products Obtained by Hydrothermal Carbonization of Palm Empty Fruit Bunches. Bioresour. Technol. 2013, 135, 683–689. 10.1016/j.biortech.2012.09.042. 23127830

[ref54] Liu Y.; He Z.; Uchimiya M. Comparison of Biochar Formation from Various Agricultural By-Products Using FTIR Spectroscopy. Mod. Appl. Sci. 2015, 9, 246–253. 10.5539/mas.v9n4p246.

[ref55] Namduri H.; Nasrazadani S. Quantitative Analysis of Iron Oxides Using Fourier Transform Infrared Spectrophotometry. Corros. Sci. 2008, 50, 2493–2497. 10.1016/j.corsci.2008.06.034.

[ref56] Tadic M.; Panjan M.; Tadic B. V.; Kopani M.; Kopanja L. Magnetic Properties of Hematite (α-Fe2O3) Nanoparticles Synthesized by Sol-Gel Synthesis Method: The Influence of Particle Size and Particle Size Distribution. J. Electr. Eng. 2019, 70, 71–76. 10.2478/jee-2019-0044.

[ref57] Ma H.; Li J. B.; Liu W. W.; Miao M.; Cheng B. J.; Zhu S. W. Novel Synthesis of a Versatile Magnetic Adsorbent Derived from Corncob for Dye Removal. Bioresour. Technol. 2015, 190, 13–20. 10.1016/j.biortech.2015.04.048. 25919932

[ref58] Ai L.; Zhang C.; Chen Z. Removal of Methylene Blue from Aqueous Solution by a Solvothermal-Synthesized Graphene/Magnetite Composite. J. Hazard. Mater. 2011, 192, 1515–1524. 10.1016/j.jhazmat.2011.06.068. 21782325

[ref59] Lafuente B.; Downs R. T.; Yang H.; Stone N. Stone N Software for Identification and Refinement of Cell Parameters from Powder Diffraction Data of Minerals Using the RRUFF Project and American Mineralogist Crystal Structure Databases. Hematite R110013, https://rruff.info/hematite/R110013 (accessed Sep 2, 2019).

[ref60] Rafatullah M.; Sulaiman O.; Hashim R.; Ahmad A. Adsorption of Methylene Blue on Low-Cost Adsorbents: A Review. J. Hazard. Mater. 2010, 177, 70–80. 10.1016/j.jhazmat.2009.12.047. 20044207

[ref61] Dinh V. P.; Huynh T. D. T.; Le H. M.; Nguyen V. D.; Dao V. A.; Hung N. Q.; Tuyen L. A.; Lee S.; Yi J.; Nguyen T. D.; et al. Insight into the Adsorption Mechanisms of Methylene Blue and Chromium(III) from Aqueous Solution onto Pomelo Fruit Peel. RSC Adv. 2019, 9, 25847–25860. 10.1039/C9RA04296B. PMC9070119 35530102

[ref62] Zhang H.; Zhang F.; Huang Q. Highly Effective Removal of Malachite Green from Aqueous Solution by Hydrochar Derived from Phycocyanin-Extracted Algal Bloom Residues through Hydrothermal Carbonization. RSC Adv. 2017, 7, 5790–5799. 10.1039/C6RA27782A.

[ref63] Gomer R. Chemisorption on Metals. Solid State Phys. 1975, 30, 93–225.

[ref64] Joo J. B.; Park J.; Yi J. Preparation of Polyelectrolyte-Functionalized Mesoporous Silicas for the Selective Adsorption of Anionic Dye in an Aqueous Solution. J. Hazard. Mater. 2009, 168, 102–107. 10.1016/j.jhazmat.2009.02.015. 19269088

[ref65] Li J.; Wang S.; Peng J.; Lin G.; Hu T.; Zhang L. Selective Adsorption of Anionic Dye from Solutions by Modified Activated Carbon. Arab. J. Sci. Eng. 2018, 43, 5809–5817. 10.1007/s13369-017-3006-0.

[ref66] Damasceno B. S.; Da Silva A. F. V.; De Araújo A. C. V. Dye Adsorption onto Magnetic and Superparamagnetic Fe3O4nanoparticles: A Detailed Comparative Study. J. Environ. Chem. Eng. 2020, 8, 103994 10.1016/j.jece.2020.103994.

[ref67] Dastgerdi Z. H.; Meshkat S. S.; Esrafili M. D. Enhanced Adsorptive Removal of Indigo Carmine Dye Performance by Functionalized Carbon Nanotubes Based Adsorbents from Aqueous Solution: Equilibrium, Kinetic, and DFT Study. J. Nanostruct. Chem. 2019, 9, 323–334. 10.1007/s40097-019-00321-0.

[ref68] Chowdhury M. F.; Khandaker S.; Sarker F.; Islam A.; Rahman M. T.; Awual M. R. Current Treatment Technologies and Mechanisms for Removal of Indigo Carmine Dyes from Wastewater: A Review. J. Mol. Liq. 2020, 318, 114061 10.1016/j.molliq.2020.114061.

[ref69] Messerle B. A.; Vuong K. Q. Synthesis of Spiroketals by Iridium-Catalyzed Double Hydroalkoxylation. Pure Appl. Chem. 2006, 78, 385–390. 10.1351/pac200678020385.

[ref70] Killian D. B.; Hennion G. F.; Nieuwland J. A. The Synthesis of Some Dioxole Derivatives from Alkylacetylenes. J. Am. Chem. Soc. 1936, 58, 1658–1659. 10.1021/ja01300a046.

[ref71] Konkol M.; Schmidt H.; Steinborn D. Iridium-Catalyzed Addition of Methanol to Internal Alkynes. J. Mol. Catal. A 2007, 261, 301–305. 10.1016/j.molcata.2006.10.027.

[ref72] Ke F.; Li Z.; Xiang H.; Zhou X. Catalytic Hydroalkoxylation of Alkenes by Iron(III) Catalyst. Tetrahedron Lett. 2011, 52, 318–320. 10.1016/j.tetlet.2010.11.036.

[ref73] Komeyama K.; Morimoto T.; Nakayama Y.; Takaki K. Cationic Iron-Catalyzed Intramolecular Hydroalkoxylation of Unactivated Olefins. Tetrahedron Lett. 2007, 48, 3259–3261. 10.1016/j.tetlet.2007.03.004.

[ref74] Alcaide B.; Almendros P.; Del Campo T. M. Chemodivergence in Alkene/Allene Cycloetherification of Enallenols: Iron versus Noble Metal Catalysis. Chem. – Eur. J. 2008, 14, 7756–7759. 10.1002/chem.200801166. 18642263

[ref75] Notar Francesco I.; Cacciuttolo B.; Pucheault M.; Antoniotti S. Simple Metal Salts Supported on Montmorillonite as Recyclable Catalysts for Intramolecular Hydroalkoxylation of Double Bonds in Conventional and VOC-Exempt Solvents. Green Chem. 2015, 17, 837–841. 10.1039/C4GC01990C.

[ref76] Santos L. L.; Ruiz V. R.; Sabater M. J.; Corma A. Regioselective Transformation of Alkynes into Cyclic Acetals and Thioacetals with a Gold(I) Catalyst: Comparison with Brønsted Acid Catalysts. Tetrahedron 2008, 64, 7902–7909. 10.1016/j.tet.2008.06.032.

[ref77] Kondolff I.; Doucet H.; Santelli M. Direct Synthesis of Protected Arylacetaldehydes by Palladium- Tetraphosphine-Catalyzed Arylation of Ethyleneglycol Vinylether. Synlett 2004, 9, 1561–1564. 10.1055/s-2004-829063.

[ref78] Zhou Y.; Xu X.; Sun H.; Tao G.; Chang X. Y.; Xing X.; Chen B.; Xu C. Development of Highly Efficient Platinum Catalysts for Hydroalkoxylation and Hydroamination of Unactivated Alkenes. Nat. Commun. 2021, 12, 1953 10.1038/s41467-021-22287-w. 33782394 PMC8007598

[ref79] Serp P.; Figueiredo J. L. Carbon Materials for Catalysis; Wiley, 2009.

[ref80] Armstrong W. D.; Rogler J. C.; Featherston W. R. Effect of Tannin Extraction on the Performance of Chicks Fed Bird Resistant Sorghum Grain Diets. Poult. Sci. 1974, 53, 714–720. 10.3382/ps.0530714.

[ref81] Liu J. F.; Zhao Z. S.; Jiang G. B. Coating Fe3O4 Magnetic Nanoparticles with Humic Acid for High Efficient Removal of Heavy Metals in Water. Environ. Sci. Technol. 2008, 42, 6949–6954. 10.1021/es800924c. 18853814

[ref82] Uddin A. B. M. H.; Khalid R. S.; Alaama M.; Abdualkader A. M.; Kasmuri A.; Abbas S. A. Comparative Study of Three Digestion Methods for Elemental Analysis in Traditional Medicine Products Using Atomic Absorption Spectrometry. J. Anal. Sci. Technol. 2016, 7, 6 10.1186/s40543-016-0085-6.

