# Supplementary material for: Hydrothermal Synthesis of Biomass-Derived Magnetic Carbon Composites for Adsorption and Catalysis
Source: ACS Omega. 2021 Nov 24;6(48):33000–9. doi: 10.1021/acsomega.1c05116 (PMC8655907; doi:10.1021/acsomega.1c05116)
Supplement: Supplementary file 1 — ao1c05116_si_001.pdf [file ao1c05116_si_001.pdf]

Supporting Information

Hydrothermal Synthesis of Biomass-Derived Magnetic Carbon Composites for Adsorption and Catalysis

Gareth Davies and James McGregor*

University of Sheffield, Department of Chemical and Biological Engineering, Mappin Street, Sheffield S1 3JD, UK

***E-mail**: james.mcgregor@sheffield.ac.uk

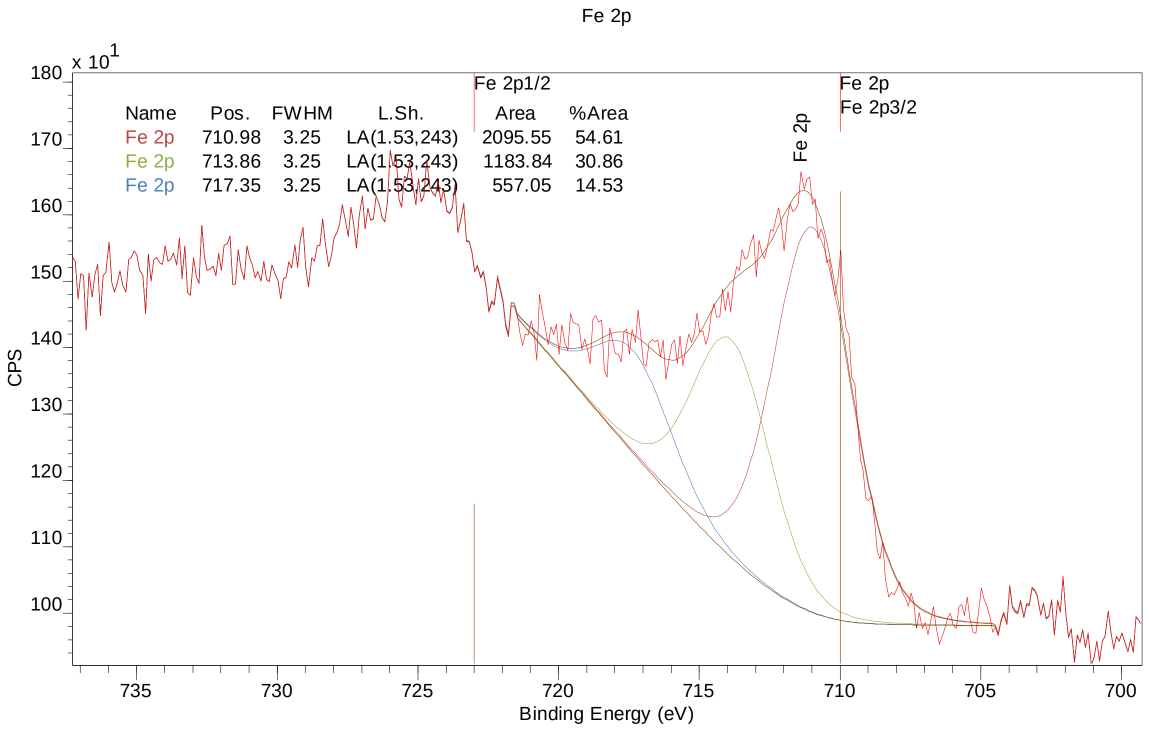

Figure S1: XPS high resolution Fe 2p from position 1, collected at 20 eV pass energy, after curve fitting to determine main peak position.

Supporting Information

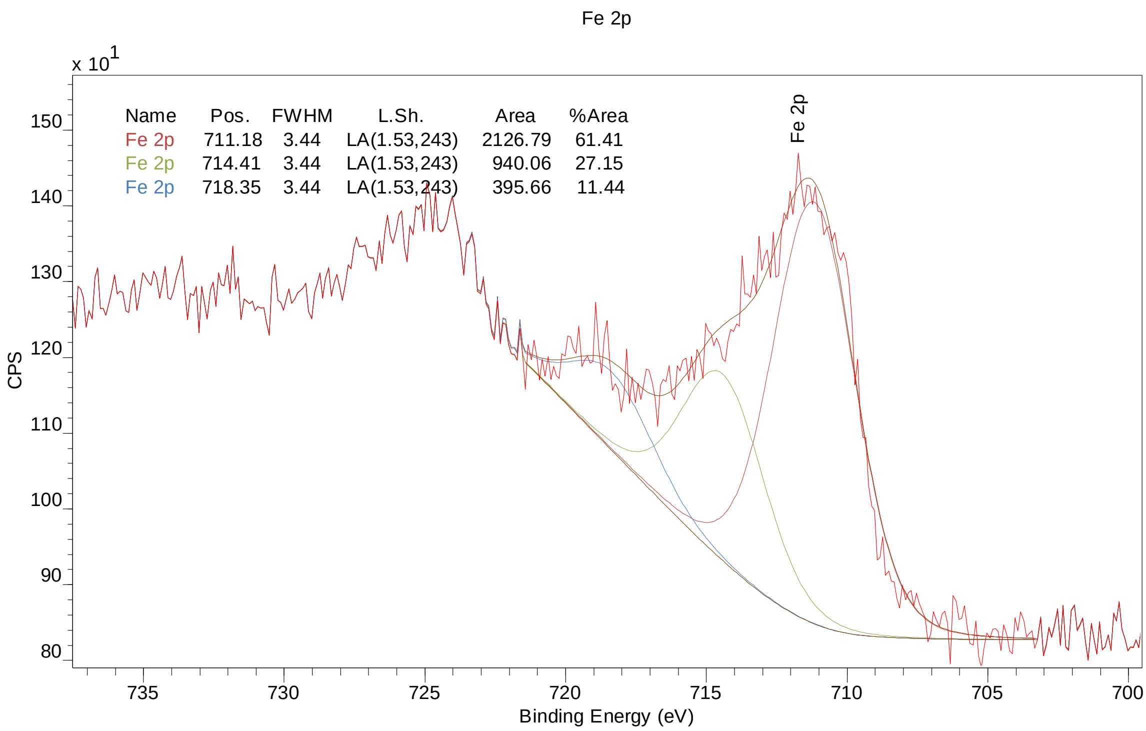

Figure S2: XPS high resolution Fe 2p from position 2, collected at 20 eV pass energy, after curve fitting to determine main peak position.

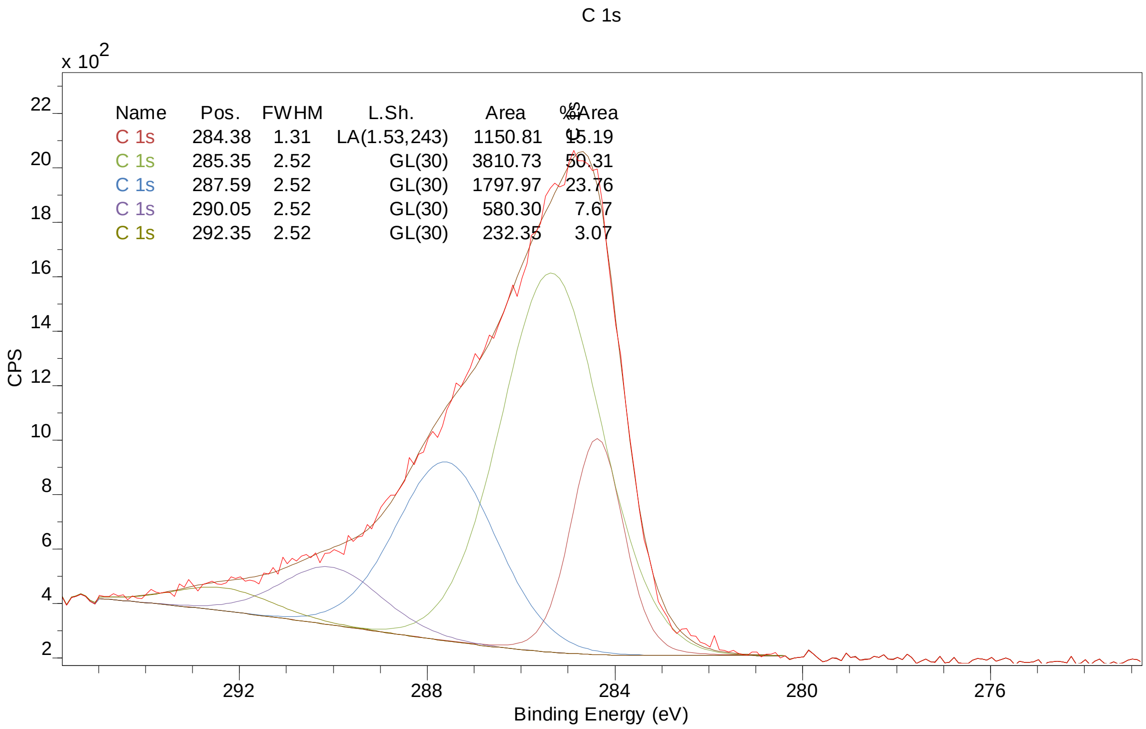

Figure S3: XPS high resolution C 1s scan from position 1, collected at 20 eV pass energy, after curve fitting.

Supporting Information

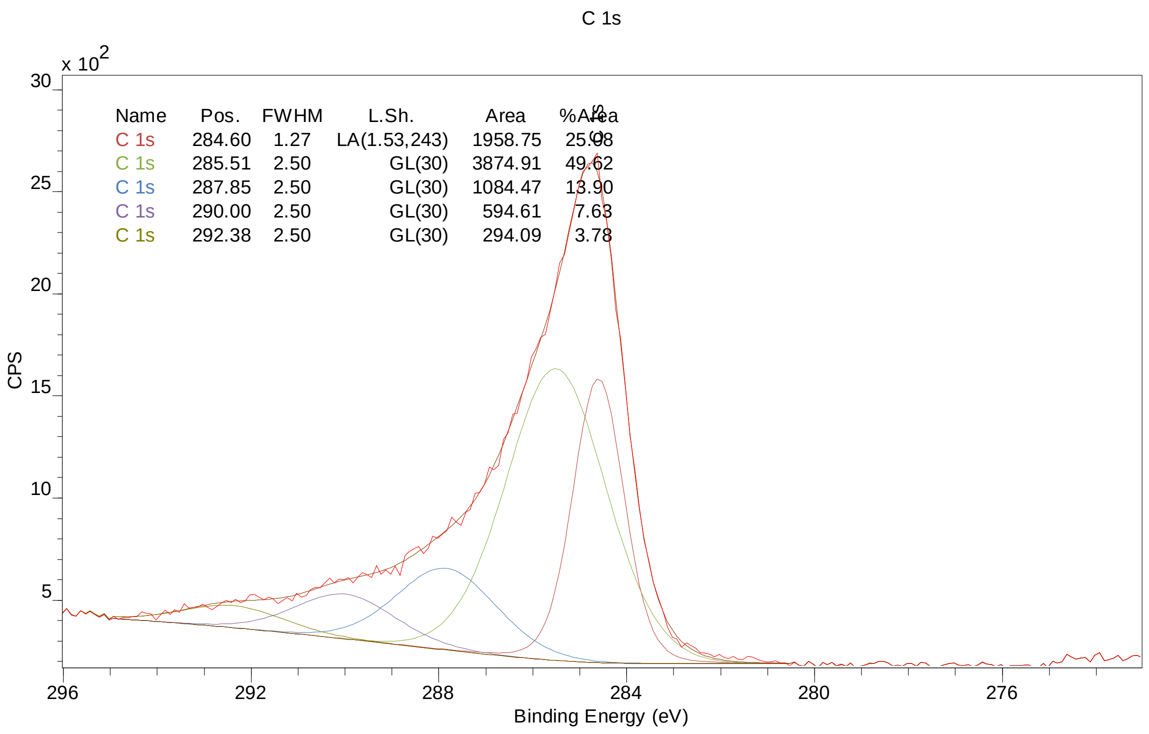

Figure S4: XPS high resolution C 1s scan from position 1, collected at 20 eV pass energy, after curve fitting.

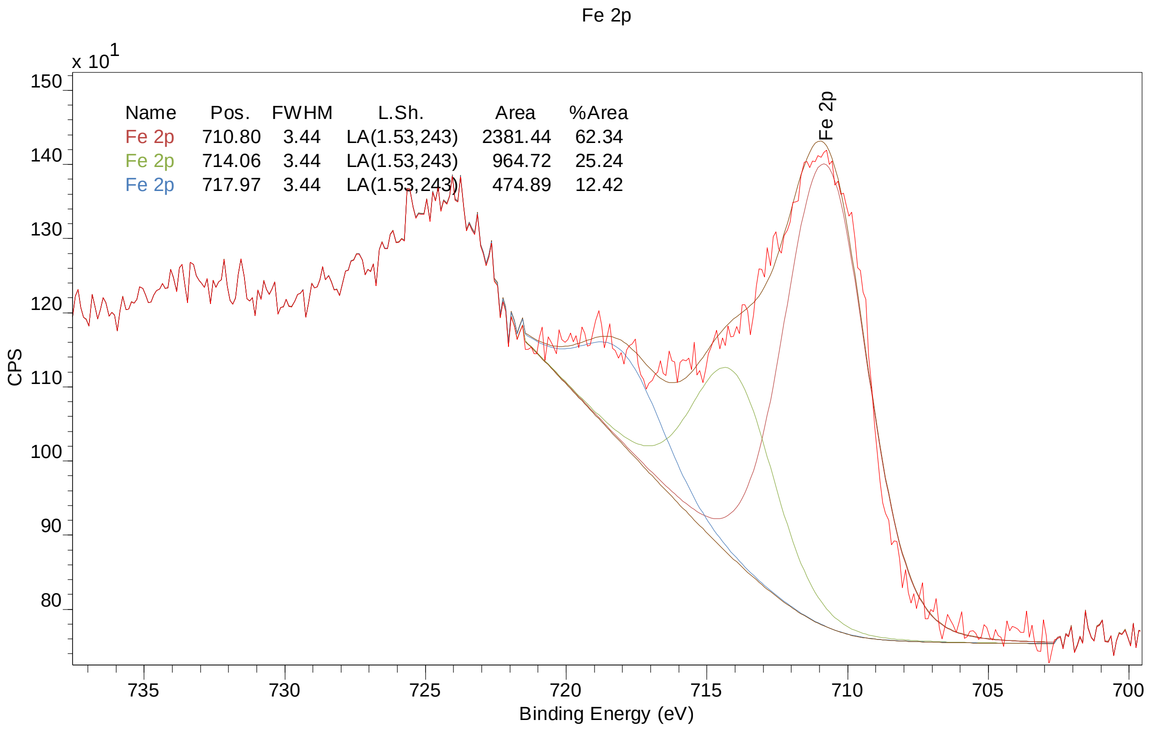

Figure S5: XPS high resolution Fe 2p from position 3, collected at 10 eV pass energy, after curve fitting to determine main peak position.

Supporting Information

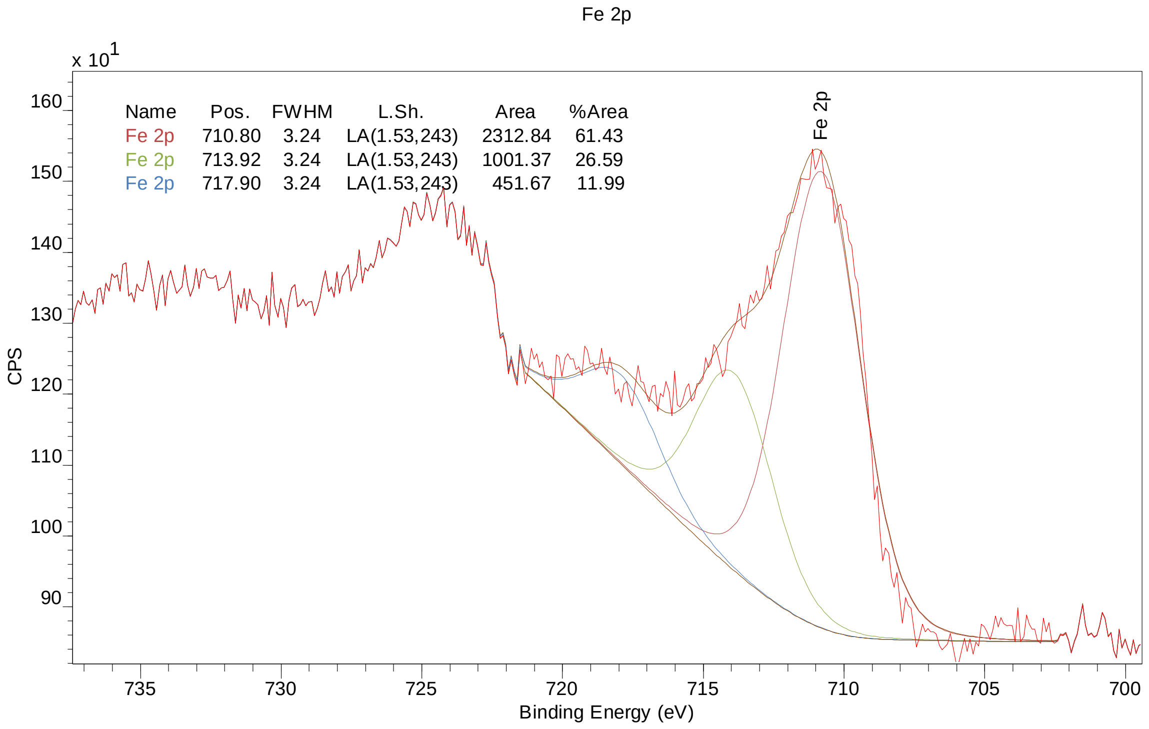

Figure S6: XPS high resolution Fe 2p from position 5, collected at 10 eV pass energy, after curve fitting to determine main peak position.

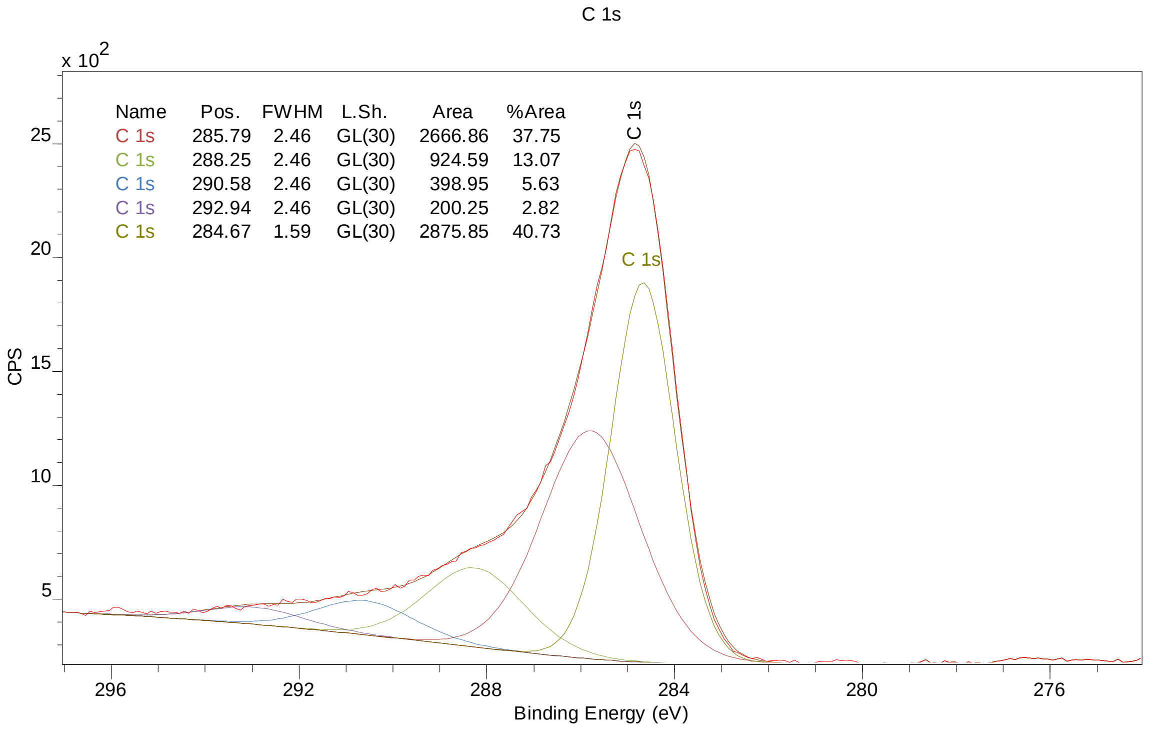

Figure S7: XPS high resolution C 1s scan from position 3, collected at 10 eV pass energy, after curve fitting.

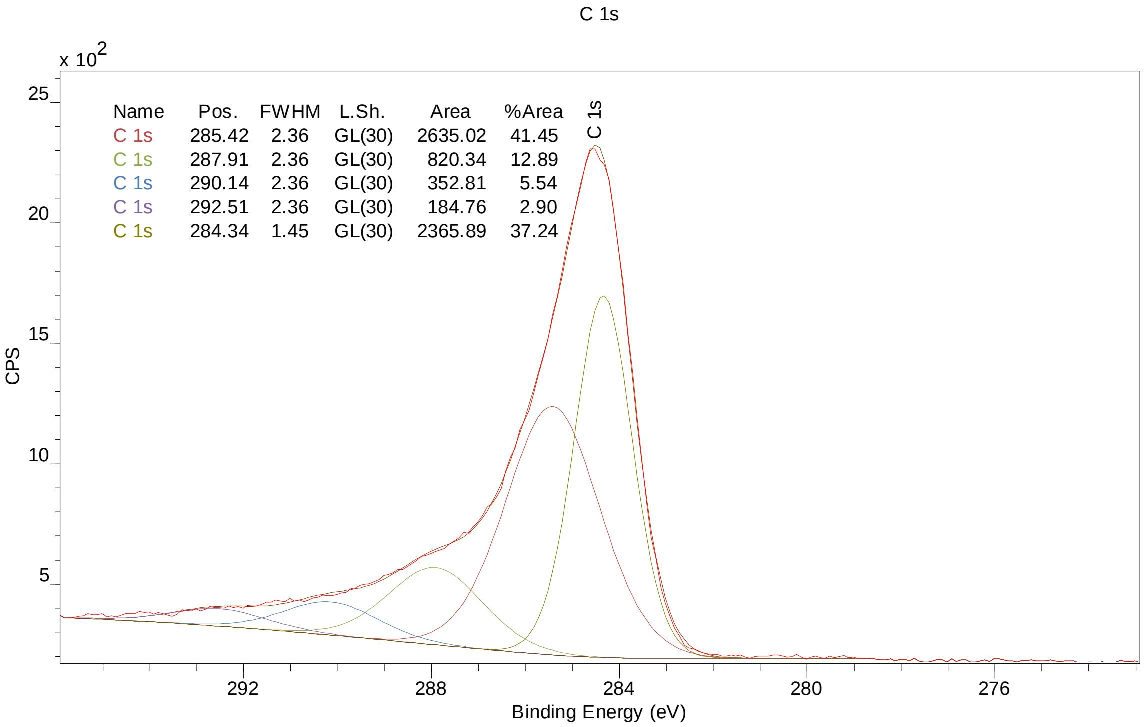

Figure S8: XPS high resolution C 1s scan from position 5, collected at 10 eV pass energy, after curve fitting.
